# 2α-Hydroxyeudesma-4,11(13)-Dien-8β,12-Olide Isolated from *Inula britannica* Induces Apoptosis in Diffuse Large B-cell Lymphoma Cells

**DOI:** 10.3390/biom10020324

**Published:** 2020-02-18

**Authors:** Dae Kil Jang, Ik-Soo Lee, Han-Seung Shin, Hee Min Yoo

**Affiliations:** 1 Department of Food Science and Biotechnology, Dongguk University, Seoul 10326, Korea; ktcommer@hanmail.net; 2 StarlingForce Co., Ltd., Seoul 08511, Korea; 3 Herbal Medicine Research Division, Korea Institute of Oriental Medicine, Daejeon 34054, Korea; knifer48@kiom.re.kr; 4 Center for Bioanalysis, Korea Research Institute of Standards and Science (KRISS), Daejeon 34113, Korea

**Keywords:** *Inula britannica*, sesquiterpene lactone, lymphoma, apoptosis, ROS

## Abstract

2α-Hydroxyeudesma-4,11(13)-dien-8β,12-olide (HEDO), a eudesmane-type sesquiterpene lactone belonging to large group of plant terpenoids isolated from *Inula britannica*, displays cytotoxic activity against diffuse large B cell lymphoma cells in vitro. However, the molecular mechanism of the anticancer effect remains unclear. In this study, we showed that HEDO inhibits cell growth by inducing apoptosis in lymphoma cell lines through its antiproliferative activity. HEDO increases the depolarization of mitochondrial membrane potential and upregulated intracellular reactive oxygen species (ROS). Furthermore, we examined the cell cycle effect, and our results provided evidence that the arrest of the cell cycle at the SubG0/G1 phase plays an important role in the ability of HEDO to inhibit cell growth in Ontario Cancer Institute (OCI)-LY3 lymphoma cells by preventing nuclear factor-kappa B (NF-κB) signaling. In addition, HEDO induced apoptosis by instigating the activation of Bcl-2-associated X (BAX) and cleaved caspase-3, decreasing B-cell lymphoma 2 (BCL2), B-cell lymphoma-extra large (BCL-XL), and procaspase 3 expression levels. Based on these findings, we suggest that HEDO has potential as an anticancer drug of lymphoma by inducing ROS-dependent accumulation of SubG0/G1 arrest and apoptosis in OCI-LY3 cells.

## 1. Introduction

Broadly, there are two major categories of lymphomas, namely Hodgkin lymphoma and non-Hodgkin lymphoma (NHL), which include a diverse set of lymphoid malignancies [1]. Diffuse large B-cell lymphoma (DLBCL) is one of the most common subtypes of NHL worldwide, comprising 30–40% of all newly diagnosed cases [2]. Gene expression profiling was used to identify three subtypes according to molecular heterogeneity: activated B cell-like (ABC), germinal center B cell-like (GCB), and primary mediastinal B cell lymphoma (PMBCL) subgroups [3,4,5]. Within the last decade, despite highly curative immunochemotherapy regimens, 30–40% of patients have relapsed/refractory disease with resistance and require salvage treatment, which does not offer satisfactory outcomes [6,7]. Among the subtypes, the least curable is activated B cell-like DLBCL, necessitating the development of new treatment strategies for this disease.

ABC-DLBCL is characterized by constitutive activation of the nuclear factor-κB (NF-κB) pathway [8]. The activation of NF-κB depends on the proteasomal degradation of the inhibitor of NF-κB (IκB) proteins, following their phosphorylation by the IκB kinase (IKK) complex [9]. NF-κB dimers are composed of p65 (also known as REL-A), REL, and p50 subunits to enter the nucleus and mediate transcription of target genes [10]. Whereas an oncogenic role for constitutive classic NF-κB activity has been demonstrated in DLBCL, inhibitors that block the activation of the canonical or alternative NF-κB pathways of DLBCL have yet to be developed.

The genus *Inula*, a variable perennial herb, is comprised of approximately 100 species worldwide that are distributed mainly in Asia, Europe, and Africa [11]. Many *Inula* species are used in traditional medicine for the treatment of bronchitis, diabetes, intestinal ulcers, digestive disorders, and inflammation in various parts of the world [12,13]. Among the species, the flowers of *Inula britannica* Linnaeus (Asteraceae) exhibit antibacterial, carminative, diuretic, and laxative properties [14], which are associated with the presence of various biologically active compounds, such as steroids, terpenoids (sesquiterpenes, diterpenes, and triterpenoids), phenolics, and flavonoids [15]. Of these, sesquiterpenes are the 15-carbon subgroup of terpenoids and the characteristic components of the *Inula* species. In the *Inula* species, most sesquiterpenes occur in a lactonized form, and the major type of sesquiterpene lactones found in this species is eudesmanolides [16]. Eudesmanolides are tricyclic sesquiterpene lactones with a five-membered γ-butyrolactone ring, which can be divided into two structural classes depending on the cyclization of the lactone ring, 6,12- and 8,12-olides.

2α-Hydroxyeudesma-4,11(13)-dien-8β,12-olide (HEDO) is a eudesmane-type sesquiterpene lactone [17], belonging to a large group of plant terpenoids, with an 8,12-olide skeleton isolated from *I. britannica*. In the course of searching for anticancer agents from natural sources, we found that HEDO exhibited dramatic cytotoxic activity against the DLBCL cell line. In the present study, we examined the anticancer activity of HEDO against OCI-LY3 cells in vitro. Furthermore, we investigated the molecular mechanism associated with the anticancer effect of HEDO.

## 2. Materials and Methods

### 2.1. Plant Material

The flowers of *I. britannica* were purchased from a traditional herbal medicine store in Daejeon, the Republic of Korea, in August 2018, and were identified by Prof. Ki Hwan Bae (College of Pharmacy, Chungnam National University, Republic of Korea). A voucher specimen (IB2018-001) has been deposited in the Herbarium of the College of Pharmacy, Chungnam National University.

### 2.2. Extraction and Isolation

The air-dried flowers of *I. britannica* (1 kg) were extracted using ethanol (10 L) at 80 °C for 3 h, then filtered and concentrated to yield an ethanol extract (60 g, 6% yield). The extract (50 g) was fractionated by Diaion HP-20 column chromatography (50 × 10 cm) and eluted with a gradient solvent system consisting of (A) methanol and (B) H_2_O. The fractions resulting from the column chromatographic separation were combined into three fractions (A–C) based on the Thin-layer chromatography (TLC) results. Among these, sesquiterpene-rich fraction B was chromatographed on a YMC reversed phase (RP)-18 column (50 × 6.5 cm) using a MeOH–H_2_O gradient solvent system (20:80→100:0) to yield three subfractions (B1–B3). Fraction B1 was further chromatographed on a YMC RP-18 column (50 × 3.5 cm) and eluted with a MeOH–H_2_O gradient solvent system (40:60→80:20) to yield HEDO (220 mg).

HEDO: Whiter powder; [α]_D_ -87 (c 0.1, MeOH); UV (MeOH) λ_max_ 207 nm; IR (KBr) ν_max_ 3479, 2980, 1745, 1645, 1320, 1262, 1141, 999, 928, and 811 cm^−1^; ^1^H-NMR (400 MHz, CD_3_OD) δ 6.20 (1H, d, *J* = 2.8 Hz, H-13a), 5.74 (1H, d, *J* = 2.8 Hz, H-13b), 4.54 (1H, m, H-8), 3.56 (1H, m, H-2), 3.16 (2H, m, H-7), 2.95 (1H, dd, *J* = 13.6, 7.6 Hz, H-6a), 2.23 (1H, dd, *J* = 13.6, 4.4 Hz, H-6b), 2.16–1.96 (2H, m, H-3), 1.84–1.73 (2H, m, H-9), 1.66 (3H, s, CH_3_-15), 1.46 (1H, dd, J = 13.6, 10.8 Hz, H-1α), 1.01 (3H, s, CH_3_-14); ^13^C-NMR (100 MHz, CD_3_OD) δ 173.0 (C-12), 141.7 (C-11), 132.0 (C-5), 127.8 (C-4), 122.8 (C-13), 77.9 (C-8), 72.8 (C-2), 41.7 (C-7), 40.5 (C-10), 38.6 (C-1), 32.1 (C-9), 28.9 (C-3), 27.8 (C-6), 21.0 (CH_3_-15), 19.1 (CH_3_-14); ESIMS *m*/*z* 249 [M + Na]^+^.

### 2.3. Cell Culture

OCI-LY3 cells were obtained from the German Collection of Microorganisms and Cell Cultures GmbH (Deutsche Sammlung von Mikroorganismen und Zellkulturen (DSMZ), Braunschweig, Germany). A culture medium for the cell line was used in accordance with the information provided by DSMZ. Cell lines were cultured in a humidified atmosphere of 5% CO_2_ at 37 °C. Subcultures were generated when the cell density reached 80–90% every 3 days.

### 2.4. Antiproliferation Assay

To assess antiproliferative effects in the presence of HEDO, cells were cultured at a cell density of 5 × 10^5^ cells per well in 100 μL advanced Roswell Park Memorial Institute (RPMI) 1640 medium. The cells were treated with different concentrations of HEDO (3, 5, 10, 20, 50, and 100 μM). Cell viability was assessed using an MTS (3-(4,5-dimethylthiazol-2-yl)-5-(3-carboxymethoxyphenyl)-2-(4-sulfophenyl)-2*H*-tetrazolium, inner salt) assay, as described in a previous paper written by the authors of this study [18].

### 2.5. Microscopy

HEDO-treated OCI-LY3 cells were examined under a phase-contrast microscope (Olympus, Tokyo, Japan) to detect morphological changes, as described in a previous paper written by the authors of this study [18].

### 2.6. Tetramethylrhodamine Methyl Ester Perchlorate (TMRM) Assay

The cells were incubated with 100 nM TMRM (Thermo Fisher Scientific, Waltham, MA, USA), which is a fluorescent indicator of the mitochondrial membrane potential. The cells were washed and resuspended in phosphate-buffered saline (PBS), measured using a flow cytometer (BD FACSVerse, BD Biosciences, San Jose, CA, USA), and analyzed using the FlowJo software.

### 2.7. Measurement of Reactive Oxygen Species (ROS) and Mitochondrial Membrane Potential (Δψm)

To measure the ROS levels in the cells, the 2′,7′-dichlorodihydrofluorescein diacetate acetyl ester (H_2_DCFDA) indicator was used (Thermo Fisher Scientific, Waltham, MA, USA). Cells were incubated with 1 μM H_2_DCFDA at room temperature. To measure the mitochondrial ROS levels in the cells, MitoSOX-red mitochondrial superoxide indicator was used (Thermo Fisher Scientific, Waltham, MA, USA). To detect Δψm, cells were incubated with MitoProbe JC-1 (5′,6,6′-tetrachloro-1,1′,3,3′-tetraethylbenzimidazolylcarbocyanine iodide) (Thermo Fisher Scientific, Waltham, MA, USA). Fluorescence-activated cell sorting (FACS) buffer (PBS supplemented with 1% fetal bovine serum) was used for washing and resuspension. Intracellular fluorescence ROS levels and Δψm were analyzed using a flow cytometer (BD FACSVerse, BD Biosciences, San Jose, CA, USA).

### 2.8. Cell Cycle Progression

Analysis of the cellular DNA content of HEDO-treated cells enables the detection of cells in each cycle phase (sub-G_0_/G_1_, G_0_/G_1_, S, or G_2_/M). HEDO-treated OCI-LY3 cells were seeded into 6-well plates and incubated for 48 h. The cells were harvested, washed with PBS, and fixed with ice-cold 80% EtOH at 4 °C for 3 h. For cell cycle analysis, the Muse cell cycle assay kit (Luminex Corporation, Austin, TX, USA) was used. The cells were evaluated using a flow cytometer (BD FACSVerse, BD Biosciences, San Jose, CA, USA), and the cell percentages of the sub-G_0_/G_1_, G_0_/G_1_, S, and G_2_/M phases were analyzed using the FlowJo software.

### 2.9. Apoptosis Analysis

The cells were analyzed by flow cytometry for phosphatidylserine detection using an Annexin V APC/PI apoptosis detection kit (BioLegend, San Diego, CA, USA). HEDO-treated and untreated OCI-LY3 cells were incubated at 37 °C for 72 h. The cells were harvested, washed with PBS, and stained with Annexin V/PI, according to the manufacturer’s instructions. After staining, the apoptotic cells were analyzed using a flow cytometer (BD FACSVerse, BD Biosciences, San Jose, CA, USA). The percentages of the cells were also analyzed using the FlowJo software.

### 2.10. Western Blot Analysis

Cells treated with HEDO were collected and lysed in a RIPA buffer containing a complete protease inhibitor cocktail. The concentrations of lysates were measured using the Bradford assay. Total protein was separated via sodium dodecyl sulfate-polyacrylamide gel electrophoresis and the gels were transferred to polyvinylidene difluoride membranes. The membranes were blocked with 5% nonfat milk. The primary antibodies, namely, anti-BCL-2, anti-BCL-XL, anti-actin (Santa Cruz Biotechnology, Dallas, TX, USA), and anti-cleaved caspase-3 (Cell Signaling Technology, Danvers, MA, USA), were diluted, according to the manufacturer’s recommendation. After treating with the primary antibodies, the membranes were incubated with secondary antibodies at 1:5000 dilutions, which were detected using an ImageQuant LAS 4000 Mini (Fujifilm, Tokyo, Japan). For the quantification, the band was analyzed using an image analysis program (Multi Gauge, Tokyo, Japan).

### 2.11. RT-qPCR

Total RNA was isolated from 48-h HEDO-treated cells using the RNeasy mini kit (Qiagen, Hilden, Germany). Each PCR reaction was performed using the iTaq Universal SYBR Green Supermix (Bio-Rad, Hercules, CA, USA). The RT-qPCR analysis was also performed on a StepOnePlus Real-Time PCR system (Thermo Fisher Scientific, Waltham, MA, USA).

### 2.12. Confocal Imaging

Cells treated with HEDO for 48 h were fixed in 4% paraformaldehyde. The fixed cells were blocked and permeabilized with 3% BSA and 0.01% Triton X-100 in PBS. p65 (Santa Cruz Biotechnology, Dallas, TX, USA) was the primary antibody used, and incubation occurred overnight at 4 °C. The secondary antibody used was donkey anti-rabbit Alexa Fluor 555 (Thermo Fisher Scientific, Waltham, MA, USA). The images were acquired using a fluorescence microscope (Olympus, Tokyo, Japan).

### 2.13. Live/Dead Fluorescence Microscopy Assay

The morphology of the OCI-LY3 cells was investigated through the fluorescent labeling of both living and dead cells using the LIVE/DEAD kit (Thermo Fisher Scientific, Waltham, MA, USA). In addition, the cells were stained in the dark using a calcein and EthD-1, according to the manufacturer’s instructions. Images were captured using a fluorescence microscope (Olympus, Tokyo, Japan).

### 2.14. Statistical Analysis

Statistical analysis was performed using GraphPad Prism 5 (GraphPad Software, Inc., San Diego, CA, USA), and the values were provided as means ± SEM. Data were further analyzed through the Student’s *t*-test, and *p*-value < 0.05 was considered statistically significant.

## 3. Results

### 3.1. Extraction and Isolation of HEDO

**Figure 1:**
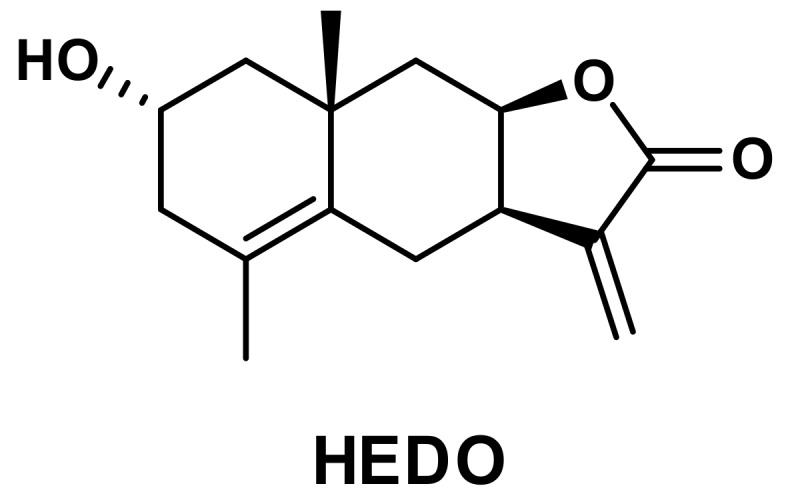
Chemical structure of 2α-Hydroxyeudesma-4,11(13)-dien-8β,12-olide (HEDO) isolated from *I. britannica* flowers.

The ethanol extract of *I. britannica* flowers was subjected to Diaion HP-20 column chromatography and divided into three fractions (A–C) based on the results of thin-layer chromatography (TLC). Fraction B was further chromatographed, leading to the isolation of a sesquiterpene lactone, which was identified as HEDO by comparing its physicochemical and spectral data to those in the literature (Figure 1) [17].

### 3.2. HEDO-Induced Antiproliferative Effect

**Figure 2:**
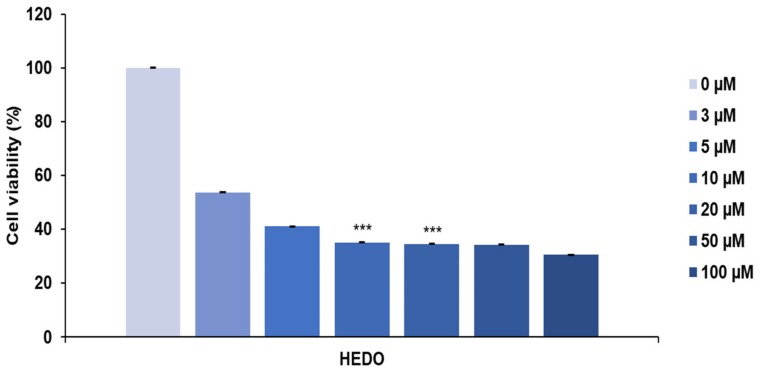
Anti-proliferative effects of *Inula britannica* HEDO on OCI-LY3 cells. Cell viability was assessed by the MTS assay at 48 h after treatment. Values indicate the means ± SEM. (n = 3, *** *p* ≤ 0.001).

The anti-proliferative effect of HEDO was tested in a cell proliferation assay using a blood cancer cell line. HEDO showed the strongest anti-proliferative activity against OCI-LY3 cells dose-dependently. The anti-proliferative effect increased with both the treatment duration and dose (Figure 2). These results indicate that HEDO has potent cytotoxic activity against OCI-LY3 cells.

### 3.3. HEDO Effects on Mitochondrial Membrane Potential

**Figure 3:**
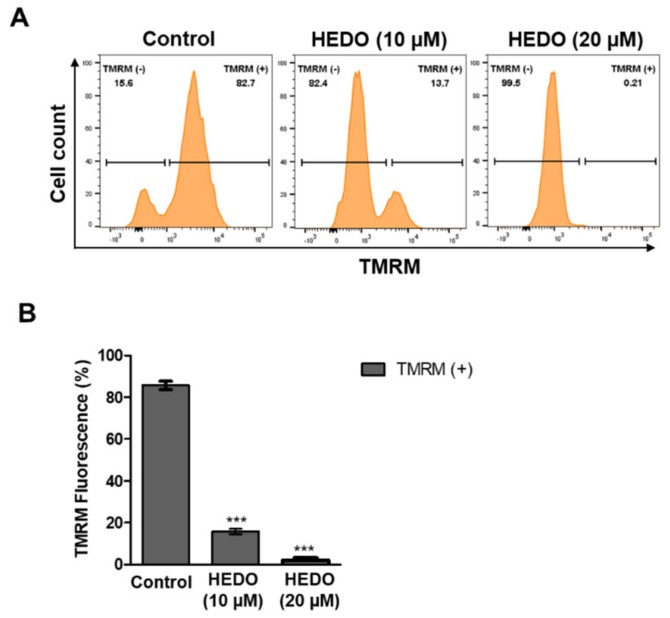
Decrease in mitochondrial membrane potential in OCI-LY3 cells due to HEDO. (**A**) Mitochondrial membrane potential of OCI-LY3 cells loaded with tetramethylrhodamine methyl ester (TMRM) (100 nM), as detected by flow cytometry. (**B**) Quantification of the membrane potential in mitochondria, as measured by flow cytometry. Values indicate the means ± SEM. (n = 3, *** *p* ≤ 0.001).

Next, the effect of HEDO on the mitochondrial membrane potential (ΔΨm) was examined by measuring tetramethylrhodamine methyl ester (TMRM) fluorescence intensity using flow cytometry. TMRM-positive quantifies the ΔΨm in lymphoma cells. HEDO-treated cells revealed a dramatic membrane potential depolarization after 48 h (Figure 3A,B). Thus, HEDO induced mitochondrial depolarization in OCI-LY3 lymphoma cells. These results suggest that HEDO is a potential chemotherapeutic agent for blood cancers.

### 3.4. HEDO-Induced Reactive Oxygen Species (ROS)

**Figure 4:**
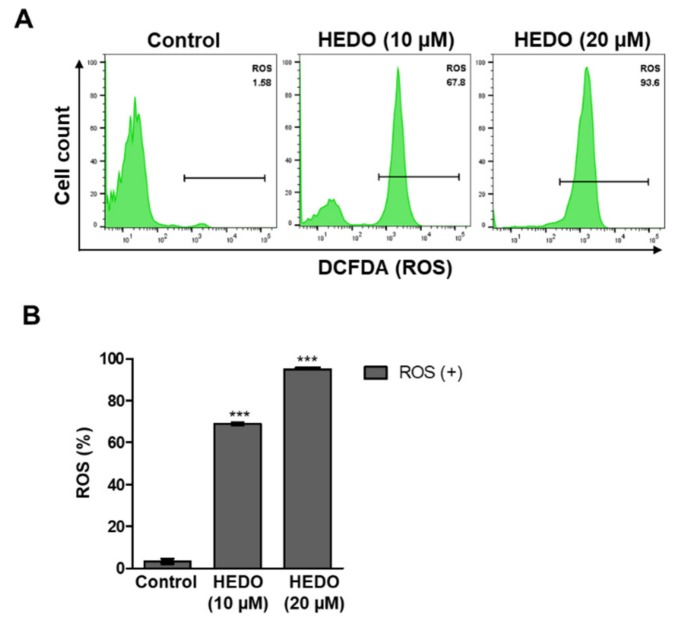
HEDO-induced changes in intracellular reactive oxygen species (ROS) levels in OCI-LY3 cells. (**A**) Measurement of ROS levels through flow cytometry with 2′,7′-dichlorodihydrofluorescein diacetate assay (H_2_DCFDA) (1 μM). (**B**) Quantified ROS levels. Values indicate the means ± SEM. (n = 3, *** *p* ≤ 0.001).

Tumor cells sustain ROS production by suppressing antioxidant levels. Tumor cells have higher levels of ROS compared with normal cells due to their elevated metabolism [19]. ROS production induced by anticancer agents is known to be associated with programmed cell death. To examine how HEDO causes OCI-LY3 cell death, the ROS levels were detected using the 2′,7′-dichlorodihydrofluorescein diacetate assay (H_2_DCFDA). Flow cytometry analysis showed that HEDO induced intracellular ROS levels in OCI-LY3 cells after 48 h (Figure 4A). ROS generation was quantified by flow cytometry, which similarly showed that ROS generation was dramatically increased by HEDO treatment (Figure 4B).

### 3.5. HEDO-Induced Cell Cycle Arrest

**Figure 5:**
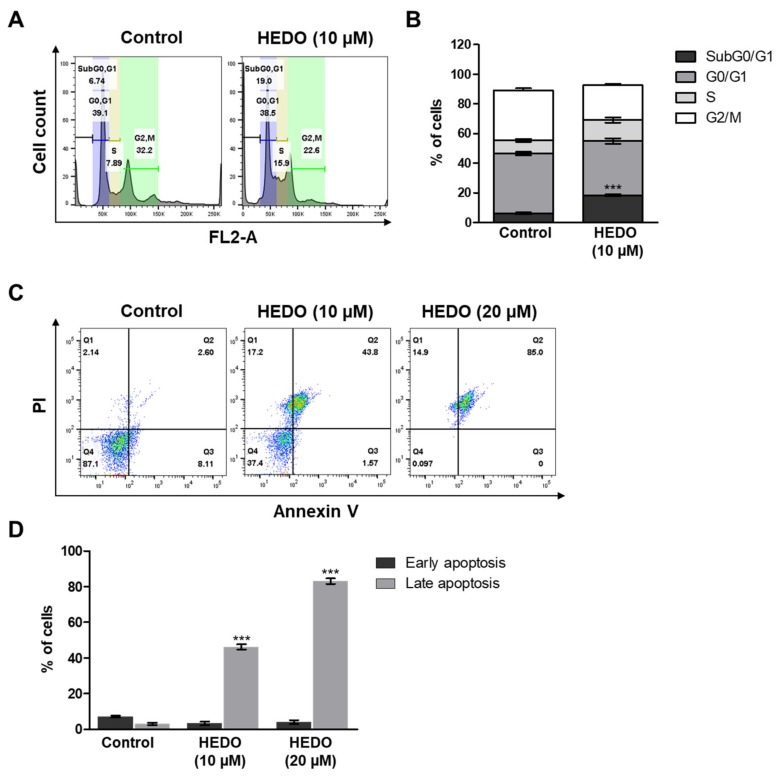
Altered cell cycle progression and apoptosis of OCI-LY3 cells due to HEDO (10 μM). (**A**) Percentage of cells in the subG0/G1, G0/G1, S, and G2/M phases after treatment with each compound. Cells were analyzed through flow cytometry. (**B**) Cell cycle analysis of cells from the same three experiments. *** *p* < 0.001 versus control group. (**C**) OCI-LY3 cells were either treated with HEDO or untreated for 72 h and apoptosis was subsequently evaluated through flow cytometry. (**D**) Quantification of apoptotic cells. Values indicate the means ± SEM. (n = 3, *** *p* ≤ 0.001).

We also found that HEDO markedly increased the sub-G_0_/G_1_ cell population. HEDO (10 μM) treatment for 48 h resulted in the accumulation of cells in the sub-G_0_/G_1_ phase, from 6.74% in the untreated control cells to 19.0% in HEDO-treated cells (Figure 5A,B). Overall, these HEDO-induced accumulation results of apoptotic lymphoma cell populations reveal that HEDO may be responsible for the prohibition of cell growth by inducing cell cycle arrest at the sub-G_0_/G_1_ phase.

### 3.6. HEDO-Induced Apoptosis

**Figure 6:**
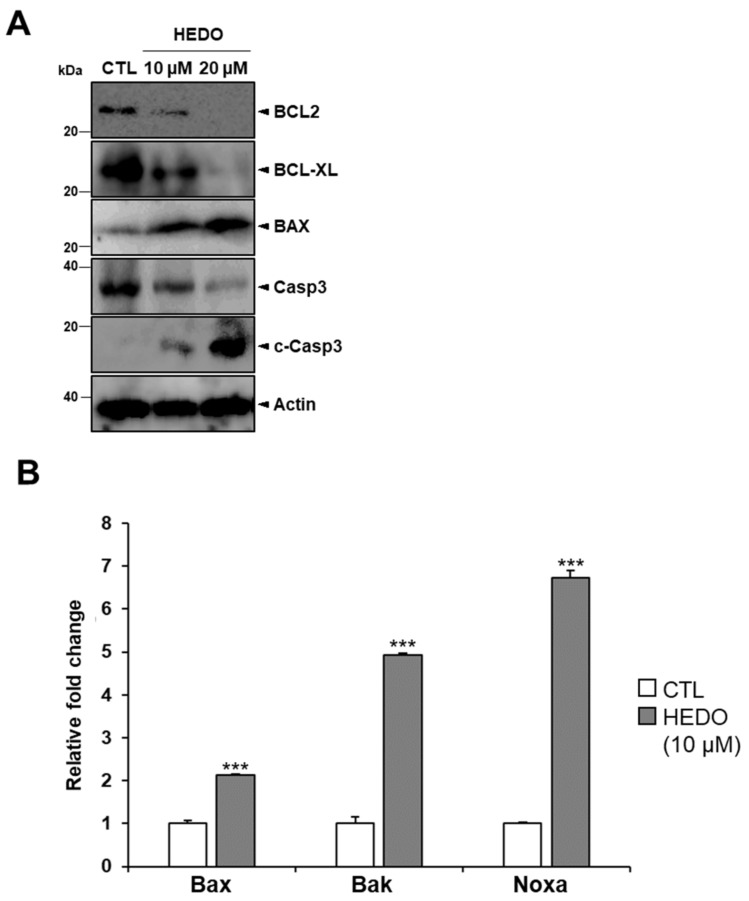
HEDO-induced apoptosis markers in OCI-LY3 cells. (**A**) Whole cell lysates from HEDO-treated cells were western blotted with antibodies specific for BCL2, BCL-XL, BAX, procaspase-3 and cleaved caspase-3 proteins. (**B**) Comparison of the qRT-PCR analysis results regarding the mRNA levels of Bax, Bak, and Noxa of the control and the HEDO**-**treated OCI-LY3 cells (n = 3, *** *p* ≤ 0.001).

HEDO-induced apoptosis was assessed using OCI-LY3 cells. The cells were stained with Annexin V and propidium iodide (PI) dyes to determine the percentages of apoptotic and viable cells. The percentages of early and late apoptotic cells increased, but the percentage of live cells decreased at 72 h post-HEDO treatment, confirming that the apoptotic cell death was induced by HEDO (Figure 5C). HEDO-treated OCI-LY3 cells showed an increased percentage of apoptotic cells within 72 h, indicating that HEDO markedly induced apoptosis in lymphoma cells (Figure 5D). Moreover, we investigated the expression levels of apoptosis-associated proteins. Western blots showed that HEDO downregulated the expression of BCL2, BCL-XL, and procaspase 3 and also upregulated BAX and cleaved caspase-3, which promotes apoptosis (Figure 6A). The qRT-PCR results demonstrated that HEDO increased pro-apoptotic markers, such as Bax, Bak, and Noxa, which promoted apoptosis in OCI-LY3 cells compared with untreated control cells (Figure 6B).

### 3.7. Inhibition Analysis of NF-κB

**Figure 7:**
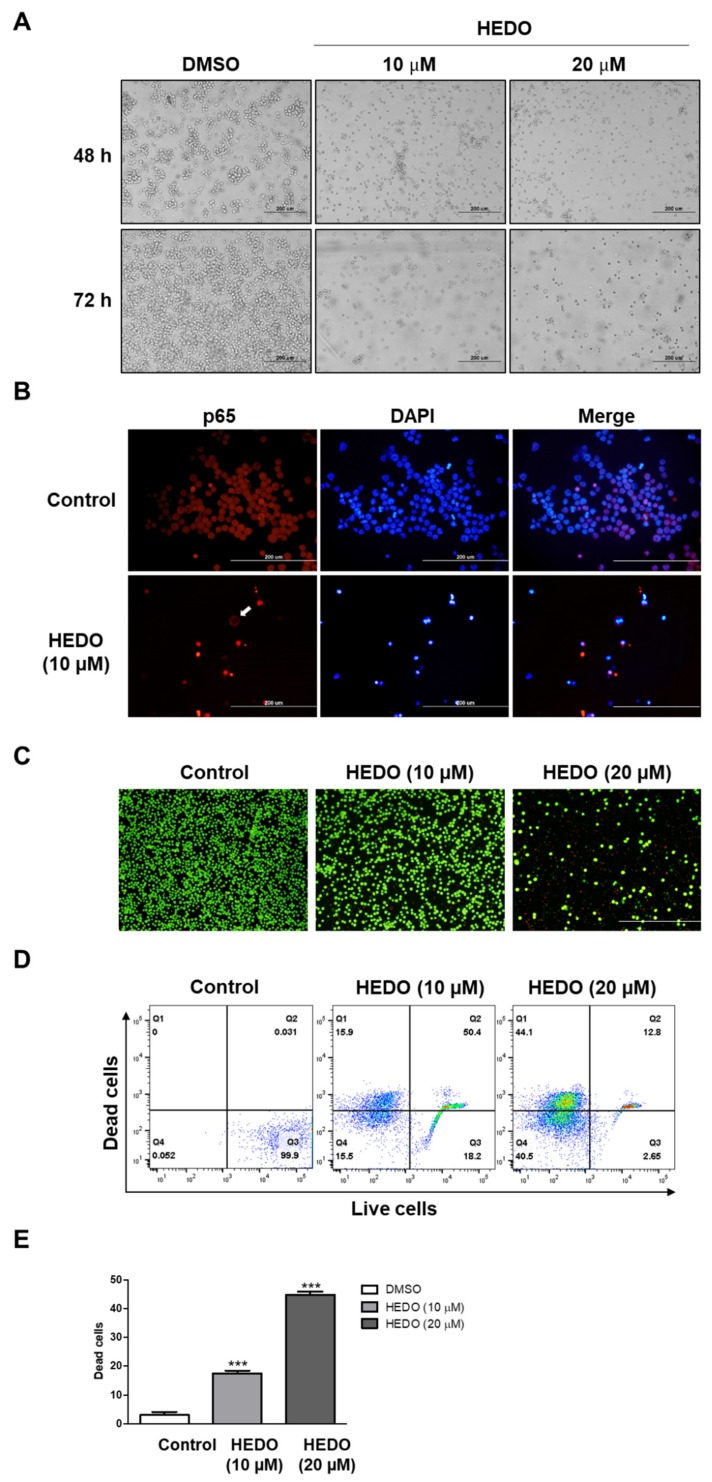
Effects of HEDO on morphological changes, p65 nuclear translocation, and cell viability in OCI-LY3 cells. (**A**) Cells treated with HEDO (10 and 20 μM) show morphological changes that are characteristic of apoptosis at 48 and 72 h post-treatment using phase contrast microscopy (n = 3, 200x magnification, scale bar: 200 μm). (**B**) Effects of HEDO on p65 nuclear translocation in OCI-LY3 cells. OCI-LY3 cells were cultured in the presence or absence of HEDO (10 µM). The white arrows show that the nuclear translocation of p65 was inhibited by HEDO and the p65 remained in the cytoplasm. (**C**) Merged fluorescence images showing untreated and HEDO-exposed (48 h) OCI-LY3 cells (green fluorescence represents live cells and red fluorescence represents dead cells). (**D**) Flow cytometry analysis of live and dead Jurkat cells with calcein and ethidium homodimer-1 staining. (**E**) Quantification of dead cells. Values indicate the means ± SEM. (n = 3, *** *p* ≤ 0.001).

Treatment with 20 μM HEDO markedly reduced cell viability, and microscopic analysis showed that HEDO treatment induced typical apoptotic morphological changes when compared with the control, dimethyl sulfoxide (DMSO)-treated cells (Figure 7A). p65 and p50 are the most common NF-κB subunits and are involved in the classical NF-κB pathway in lymphoma [20]. Nuclear localization of p65 was used as a measure of NF-κB activation [21]. In this study, HEDO was able to promote apoptosis by inhibiting the nuclear translocation of p65 (Figure 7B).

### 3.8. Live/Dead Assay in OCI-LY3 Cells

The viability/cytotoxicity assay is commonly used to quantify live and dead cell populations, which involves labeling the cells with ethidium homodimer-1 (EthD-1, red) and calcein (green) fluorophores. The live/dead assay was imaged using a fluorescence microscope (Figure 7C). Upon examining the characteristics of the live and dead stains, it was found that the HEDO (10 μM)-treated population showed weak red fluorescence. OCI-LY3 cells exposed to 20 μM HEDO demonstrated a dramatic increase in red fluorescence-labeled dead cells within 48 h of treatment. However, this observation was the reverse in the DMSO control (Figure 7D,E). These results support the results mentioned above that HEDO dramatically increased the proportion of apoptotic cells.

## 4. Discussion

NHL is the most common hematologic malignancy, with more than 385,000 incidences occurring annually worldwide. The estimated number of annual worldwide deaths due to NHL exceeds 199,000 [22]. Although most patients with DLBCL are cured through existing immunotherapy and chemotherapy regimens, approximately 20–30% of patients are refractory to chemotherapy or suffer relapses after salvage chemotherapy and stem cell transplantation [23]. Historically, natural products from plants and animals have played an important role as cancer chemotherapeutic agents in humans [24]. More recently, natural products have continued to enter clinical trials and provide leads for compounds undergoing clinical trials as anticancer agents. Therefore, natural products play a crucial role in the discovery of new anticancer drugs [25].

It is well documented that medicinal plants exhibit notable anticancer activity against various tumors [26]. Plants contain abundant compounds that consistently reduce the risk of cancer in almost all parts of the body, such as the lungs, colon, rectum, prostate, cervix, stomach, pancreas, breasts, and bladder [27]. Therefore, efforts are being made to develop anticancer substances derived from natural products that can prevent and slow down the initial and subsequent development of cancer [28]. Sesquiterpenes and their derivatives are large groups of naturally occurring compounds spread throughout the plant kingdom. Various plants used in traditional medicine contain large amounts of these compounds, but almost all are exclusively found in the Asteraceae family [29]. Such compounds exhibit a variety of pharmacological activities, including cytotoxic and antitumor activities, and consequently, plants containing these compounds are promising ingredients for novel anticancer candidate drugs of natural origin [30]. *Inula britannica* is an erect, sunflower-like, herbaceous biennial or perennial plant in the Asteraceae family and its flowers are used in traditional Chinese medicine for the treatment of various diseases, such as digestive disorders, bronchitis, and inflammation [31]. This plant has been shown to contain high levels of sesquiterpenes, and some of these sesquiterpenes, especially sesquiterpene lactones, have been reported to exhibit potent cytotoxic and apoptotic activities [14,15].

Eudesmanolides are a class of sesquiterpene lactones most widely found in *Inula* species containing more than 200 members, many of which exhibit antifungal, antibacterial, and anticancer activities [15,16]. Such tricyclic terpenoids are characterized by a 1,4α-dimethyldecahydronaphthalene framework with a five-membered γ-butyrolactone ring, and most incorporate a *trans*-fused decalin system. HEDO is a sesquiterpene lactone with an eudesmanolide skeleton, which was first found in *Pulicaria undulata* C.A. Mey., belonging to the Asteraceae family [17]. To the best of our knowledge, this is the first report of the isolation of HEDO from *I. britannica*. With the exception of its nitric oxide production inhibitory effect in LPS-induced RAW 264.7 cells [32], little is known about the biological activity of this compound to date.

In this study, we sought to evaluate the anticancer activity of HEDO, a eudesmane-type sesquiterpene lactone isolated from *I. britannica*, against DLBCL cells in vitro. We found that HEDO inhibits OCI-LY3 cell growth dose-dependently from 0 to 20 μM. However, only a slight effect was observed with 50 and 100 μM. As cancer is a multifactorial disease, it requires treatment with compounds able to target multiple intracellular molecules [33,34]. Traditional medicines and natural products are used as direct sources of natural agents that harbor multiple therapeutic effects [24,35]. Thus, researchers began to investigate the anti-tumor activity of natural compounds and subsequently strived to understand the mechanisms of the actions involved [36]. HEDO appears to have multiple molecular targets related to proteins involved in cell death or cell survival. On a different note, the pharmacokinetic variability of drugs often limits optimal effectiveness due to toxic side effects [37]. Considering that cancer treatment using HEDO of high doses has less toxic effects, it has a potential advantage in the development of drugs with optimal doses [38,39].

Next, we performed both MMP and ROS assays with two time points (6 and 12 h). The 10 μM HEDO-treated cells revealed an initiation of membrane potential depolarization even after the short time periods of 6 and 12 h (Supplementary Figure S1). Moreover, the result of the ROS experiment showed that the treatment of HEDO (10 μM) over a short time also markedly induced ROS in a time-dependent manner (Supplementary Figure S2), which reached a maximum after 48 h. It is important to determine whether HEDO acts as an oxidant or antioxidant as both can induce apoptosis. Uncontrolled production of oxidants results in oxidative stress that impairs cellular functions and contributes to the development of cancer [40]. ROS play an important role in the maintenance of the redox balance [41]. Oxidants are primarily responsible for generating excess cellular levels of ROS that causes damage to nucleic acids, proteins, lipids, and organelles, which can lead to the activation of cell death processes, such as apoptosis [42,43]. It has been long thought that antioxidants reduce ROS produced in normal cellular processes and protect cells from oxidative damage. However, a recent study indicated that antioxidants cause direct damage to DNA; moreover, double-strand breaks (DSBs) of DNA in cells are difficult to repair and are directly related to genetic mutation, apoptosis, and cancer initiation [44]. Another example is that Luteolin induces apoptotic cell death via antioxidant activity in human colon cancer cells [45]. To test whether HEDO is an oxidant or antioxidant, the total cellular ROS level was measured using H_2_DCFDA dye after HEDO treatment. Our data showed that the total cellular level of ROS was dramatically increased by HEDO treatment (Figure 4B). Moreover, we performed flow cytometry analysis by using the fluorescent dye MitoSOX-Red Mitochondrial Superoxide Indicator [46]. We demonstrated that mitochondrial ROS was dramatically enhanced by HEDO and the increased mitochondrial ROS was reduced by an ROS scavenger [47], ascorbic acid (AA) (Supplementary Figure S5A,B). These data indicate that HEDO serves as an oxidant that generates mitochondrial ROS. One of the first stages of apoptosis involves changes seen in mitochondria, such as membrane potential collapses [48]. JC-1 is a cell-permeant lipophilic cationic dye that accumulates in mitochondria and emits a red fluorescence color in healthy, active mitochondria due to the formation of aggregates [49]. As the membrane potential collapses, the aggregates fall apart, and the fluorescence shifts to a green color. Therefore, a reduction in the red/green fluorescence intensity ratio can indicate the depolarization of mitochondria occurring during apoptosis [50]. The percentage of cells with JC-1 green fluorescence significantly increased in OCI-LY3 cells, and a loss of mitochondrial membrane potential (ΔΨm) was observed under HEDO treatment (Supplementary Figure S6A,B). These effects were reduced by an ROS scavenger, ascorbic acid (AA). All of these results suggest that HEDO acts as an oxidant that stimulates the apoptosis of OCI-LY3 cells through increased mitochondrial ROS, decreased mitochondrial membrane potential, and subsequent damage to mitochondria. Elevated oxidative stress observed in cancer cells can result not only from ROS overproduction but also from low levels or inactivation of antioxidant mechanisms [51,52,53]. Our data showed that the level of ROS was dramatically increased by HEDO treatment (Figure 4B). To determine the molecular mechanism, we performed a qRT-PCR experiment using antioxidant-related genes. qRT-PCR analysis revealed that the HEDO treatment condition significantly decreased the mRNA expression level of antioxidant genes such as SOD1, GPx, and CAT (Supplementary Figure S7). Therefore, the data indicate that HEDO serves as an oxidant that downregulates antioxidants, which subsequently leads to apoptosis caused by the accumulated ROS. In addition, the ability of oxidative stress (which is due to an excessive accumulation of ROS) to provoke cell death through massive cellular damage associated with lipid peroxidation and alterations of proteins and nucleic acids has been well reported [41]. Notably, ROS accumulation preceded mitochondrial membrane alterations and other typical apoptotic events [54]. Likewise, the treatment of HEDO leads to ROS accumulation that causes OCI-LY3 lymphoma cell death.

To prove ROS-mediated apoptosis induced in OCI-LY3 lymphoma cells, we performed an apoptosis assay using PI/annexin V-APC for FACS analysis after 24 and 48 h using the OCI-LY3 cell line (Supplementary Figure S3). The apoptosis analysis results showed that HEDO (10 μM) treatment for 24 and 48 h also markedly induced the percentages of early and late apoptotic cells in a time-dependent manner, which reached a maximum after 72 h. Many types of cancer cells exhibit increased levels of ROS, and ROS production has been proven to be associated with apoptosis caused by anticancer agents [55]. Accordingly, an accumulation of ROS corresponds to apoptosis, suggesting its importance in the anticancer activity of HEDO. In this study, our novel discovery demonstrates that HEDO is a potent agent that induces apoptosis in lymphoma cells through an ROS-dependent pathway.

Recent studies of apoptosis in cancer cells have led to the discovery of several molecules which act according to both the extrinsic and intrinsic apoptotic pathways [56,57]. The extrinsic pathway provokes apoptosis through a caspase cascade that eventually leads to cell death [58]. On the other hand, the intrinsic apoptotic pathway is mitochondria-dependent and responds to different stress conditions such as genetic damage, cytosolic calcium, and oxidative stress [41,59]. These types of internal stimuli initiate pro-apoptotic members of the BCL-2 family and induce the interruption of mitochondrial membrane permeability (MMP), which is characterized by a decrease in mitochondria membrane potential [60]. Anti-apoptotic proteins such as BCL-2 and BCL-XL ensure survival by preventing the activation of pro-apoptotic proteins like BAX, which is involved in the formation of large macro-pores in the mitochondrial outer membrane [57,61]. In addition, BAX signaling activates the effector caspase, caspase-3, which triggers apoptosis [62]. To investigate the signaling molecule for the confirmation of the intrinsic (or mitochondrial) signaling pathway of apoptosis, we performed western blot analysis with two concentrations (10 and 20 μM) of HEDO. The western blots showed that HEDO not only downregulated the expression of BCL2, BCL-XL, and procaspase 3, but also upregulated BAX and cleaved caspase-3, which promotes apoptosis (Figure 6). Therefore, HEDO is a potential anticancer drug that promotes the intrinsic apoptotic pathway for lymphoma.

NF-κB is a critical transcription factor that regulates multiple genes associated with tumorigenesis in DLBCL. Thus, we investigated the relationship of this signaling and how it defines the molecular mechanism underlying HEDO-induced cell apoptosis. One function of NF-κB is the promotion of cell survival through the induction of target genes, whose products inhibit components of apoptotic machinery in cancer cells [63]. NF-κB induces the expression of Bcl-2 family members, such as Bcl-XL, a process that prevents apoptosis by inhibiting permeability transition and the depolarization of mitochondria [64,65]. We performed western blot analysis with two concentrations (10 and 20 μM) of HEDO. HEDO induces cell apoptosis in OCI-LY3 cells through dose-dependent downregulation of phosphorylated-IκBα (p-IκBα) (Supplementary Figure S4), which is related to NF-κB signaling, a critical regulator for the transcription factor p65 that regulates multiple genes associated with tumorigenesis. Nucleocytoplasmic shuttling of p65 is crucial for the transcriptional regulation of target genes [66]. Recently, several strategies have been investigated for the development of inhibitors that prevent p65 nuclear translocation [67]. For example, the adapter protein importin enables p65 translocation through the nuclear pore complex (NPC) [68]. This suggests that inhibitors of p65–importin adapter interactions could be effective and selective NF-κB inhibitors [68]. The data indicated that the nuclear translocation of p65 was inhibited by HEDO and the p65 remained in the cytoplasm (Figure 7B). This result suggests that HEDO has therapeutic potential in inhibiting NF-κB.

## 5. Conclusions

In this study, we subjected OCI-LY3 cells to anti-proliferation and apoptotic effects in vitro and detected that HEDO induced decreased levels of BCL2, BCL-XL, and procaspase 3, an upregulation of BAX and cleaved caspase-3, and increased the expression of ROS. In addition, treatment with HEDO increased the number of apoptotic cells and displayed potent mitochondrial depolarization effects in OCI-LY3 cells. These findings suggest that HEDO could be a potential multi-target therapeutic agent for lymphoma.

## Supplementary Materials

The following are available online at https://www.mdpi.com/2218-273X/10/2/324/s1, Figure S1: HEDO reduced the mitochondrial membrane potential within a short time in OCI-LY3 cells. Mitochondrial membrane potential of OCI-LY3 cells after 6 and 12 h loaded with TMRM (100 nM), as detected by flow cytometry, Figure S2: HEDO induced intracellular ROS levels within a short time in OCI-LY3 cells. Measurements of ROS levels in HEDO-treated OCI-LY3 cells after 6 and 12 h, as detected by flow cytometry with DCFDA (1 µM), Figure S3: HEDO-induced apoptosis after 24 h and 48 h in OCI-LY3 cells. OCI-LY3 cells were untreated or treated with HEDO for 24 and 48 h. Afterwards, apoptosis evaluation was performed via Annexin V-APC/PI double staining and flow cytometry, Figure S4: Effects of HEDO on NF-κB signaling in OCI-LY3 cells. Whole cell lysates were used to determine the expression levels of phosphorylated-I kappa B alpha (p-IκBα) and IκBα after treating the cells with HEDO, Figure S5: HEDO induced mitochondrial ROS levels in OCI-LY3 cells. (A) Measurements of mitochondrial ROS levels in HEDO-treated OCI-LY3 cells after 24 h, as detected by flow cytometry with MitoSOX (5 µM). (B) Quantified mitochondrial ROS levels. Values indicate the means ± SEM. (n = 3, * *p* < 0.05, *** *p* ≤ 0.001), Figure S6: Mitochondrial membrane potential changes induced by HEDO in OCI-LY3 cells. (A) Measurements of mitochondrial membrane potential in HEDO-treated OCI-LY3 cells after 24 h, as detected by flow cytometry with JC-1 (2 µM). (B) Quantified mitochondrial ROS levels, Figure S7: Effect of HEDO on the mRNA expression levels of involved antioxidants in OCI-LY3 cells. The qRT-PCR analysis results of the mRNA levels of SOD1, GPx, and CAT in the control and the HEDO (10 μM)-treated OCI-LY3 cells (n = 3, *** *p* ≤ 0.001) were compared.

## References

[1] Shaffer A.L., Young R.M., Staudt L.M. (2012). Pathogenesis of Human B Cell Lymphomas. Annu. Rev. Immunol..

[2] Siegel R.L., Miller K.D., Jemal A. (2019). Cancer statistics, 2019. CA. Cancer J. Clin..

[3] Rosenwald A., Wright G., Leroy K., Yu X., Gaulard P., Gascoyne R.D., Chan W.C., Zhao T., Haioun C., Greiner T.C. (2003). Molecular diagnosis of primary mediastinal B cell lymphoma identifies a clinically favorable subgroup of diffuse large B cell lymphoma related to Hodgkin lymphoma. J. Exp. Med..

[4] Alizadeh A.A., Elsen M.B., Davis R.E., Ma C.L., Lossos I.S., Rosenwald A., Boldrick J.C., Sabet H., Tran T., Yu X. (2000). Distinct types of diffuse large B-cell lymphoma identified by gene expression profiling. Nature.

[5] Miao Y., Medeiros L.J., Li Y., Li J., Young K.H. (2019). Genetic alterations and their clinical implications in DLBCL. Nat. Rev. Clin. Oncol..

[6] Lees C., Keane C., Gandhi M.K., Gunawardana J. (2019). Biology and therapy of primary mediastinal B-cell lymphoma: Current status and future directions. Br. J. Haematol..

[7] Young R.M., Phelan J.D., Wilson W.H., Staudt L.M. (2019). Pathogenic B-cell receptor signaling in lymphoid malignancies: New insights to improve treatment. Immunol. Rev..

[8] Compagno M., Lim W.K., Grunn A., Nandula S.V., Brahmachary M., Shen Q., Bertoni F., Ponzoni M., Scandurra M., Califano A. (2009). Mutations of multiple genes cause deregulation of NF-B in diffuse large B-cell lymphoma. Nature.

[9] Taniguchi K., Karin M. (2018). NF-B, inflammation, immunity and cancer: Coming of age. Nat. Rev. Immunol..

[10] Karin M., Greten F.R. (2005). NF-κB: Linking inflammation and immunity to cancer development and progression. Nat. Rev. Immunol..

[11] Seca A.M.L., Grigore A., Pinto D.C.G.A., Silva A.M.S. (2014). The genus Inula and their metabolites: From ethnopharmacological to medicinal uses. J. Ethnopharmacol..

[12] Zhao Y.M., Zhang M.L., Shi Q.W., Kiyota H. (2006). Chemical constituents of plants from the Genus Inula. Chem. Biodivers..

[13] Bai N., Zhou Z., Zhu N., Zhang L., Quan Z., He K., Zheng Q.Y., Ho C.-T. (2005). Antioxidative flavonoids from the flower of Inula britannica. J. Food Lipids.

[14] Bai N., Lai C.-S., He K., Zhou Z., Zhang L., Quan Z., Zhu N., Zheng Q.Y., Pan M.-H., Ho C.-T. (2006). Sesquiterpene Lactones from Inula b ritannica and Their Cytotoxic and Apoptotic Effects on Human Cancer Cell Lines. J. Nat. Prod..

[15] Khan A.L., Hussain J., Hamayun M., Gilani S.A., Ahmad S., Rehman G., Kim Y.H., Kang S.M., Lee I.J. (2010). Secondary metabolites from Inula britannica L. and their biological activities. Molecules.

[16] Wang G.W., Qin J.J., Cheng X.R., Shen Y.H., Shan L., Jin H.Z., Zhang W.D. (2014). Inula sesquiterpenoids: Structural diversity, cytotoxicity and anti-tumor activity. Expert Opin. Investig. Drugs.

[17] Rustaiyan A., Habibi Z., Saberi M., Jakupovic J. (1991). A nor-guaianolide and A glaucolide-like eudesmanolide from Pulicaria undulata. Phytochemistry.

[18] Thuy N.T.T., Lee J.-E., Yoo H.M., Cho N. (2019). Antiproliferative Pterocarpans and Coumestans from *Lespedeza bicolor*. J. Nat. Prod..

[19] Nogueira V., Hay N. (2013). Molecular pathways: Reactive oxygen species homeostasis in cancer cells and implications for cancer therapy. Clin. Cancer Res..

[20] Zhang M., Xu-Monette Z.Y., Li L., Manyam G.C., Visco C., Tzankov A., Wang J., Montes-Moreno S., Dybkaer K., Chiu A. (2016). RelA NF-kB subunit activation as a therapeutic target in diffuse large B-cell lymphoma. Aging (Albany. N. Y.).

[21] Hussain A.R., Uddin S., Ahmed M., Al-Dayel F., Bavi P.P., Al-Kuraya K.S. (2013). Phosphorylated IκBα Predicts Poor Prognosis in Activated B-Cell Lymphoma and Its Inhibition with Thymoquinone Induces Apoptosis via ROS Release. PLoS ONE.

[22] Ghobadi A. (2018). Chimeric antigen receptor T cell therapy for non-Hodgkin lymphoma. Curr. Res. Transl. Med..

[23] Friedberg J.W. (2011). Relapsed/refractory diffuse large B-cell lymphoma. Hematology Am. Soc. Hematol. Educ. Program..

[24] Dias D.A., Urban S., Roessner U. (2012). A Historical overview of natural products in drug discovery. Metabolites.

[25] Harvey A.L., Edrada-Ebel R., Quinn R.J. (2015). The re-emergence of natural products for drug discovery in the genomics era. Nat. Rev. Drug Discov..

[26] Dai J., Mumper R.J. (2010). Plant Phenolics: Extraction, Analysis and Their Antioxidant and Anticancer Properties. Molecules.

[27] Steinmetz K.A., Potter J.D. (1991). Vegetables, fruit, and cancer. I. Epidemiology. Cancer Causes Control..

[28] Chuang S.E. (2000). Curcumin-containing diet inhibits diethylnitrosamine-induced murine hepatocarcinogenesis. Carcinogenesis.

[29] Seca A.M.L., Pinto D.C.G.A., Silva A.M.S. (2015). Metabolomic Profile of the Genus Inula. Chem. Biodivers..

[30] Zhang S., Won Y.K., Ong C.N., Shen H.M. (2005). Anti-cancer potential of sesquiterpene lactones: Bioactivity and molecular mechanisms. Curr. Med. Chem. - Anti-Cancer Agents.

[31] Tang S.A., Zhu H., Qin N., Zhou J.Y., Lee E., Kong D.X., Jin M.H., Duan H.Q. (2014). Anti-inflammatory terpenes from flowers of Inula japonica. Planta Med..

[32] Zhang S.D., Qin J.J., Jin H.Z., Yin Y.H., Li H.L., Yang X.W., Li X., Shan L., Zhang W.D. (2012). Sesquiterpenoids from Inula racemosa Hookf. Inhibit nitric oxide production. Planta Med..

[33] Chamberlin S.R., Blucher A., Wu G., Shinto L., Choonoo G., Kulesz-Martin M., McWeeney S. (2019). Natural product target network reveals potential for cancer combination therapies. Front. Pharmacol..

[34] Russo M., Spagnuolo C., Tedesco I., Russo G.L. (2010). Phytochemicals in cancer prevention and therapy: Truth or dare?. Toxins (Basel)..

[35] Yuan H., Ma Q., Ye L., Piao G. (2016). The traditional medicine and modern medicine from natural products. Molecules.

[36] Lichota A., Gwozdzinski K. (2018). Anticancer Activity of Natural Compounds from Plant and Marine Environment. Int. J. Mol. Sci..

[37] Aung T.N., Qu Z., Kortschak R.D., Adelson D.L. (2017). Understanding the effectiveness of natural compound mixtures in cancer through their molecular mode of action. Int. J. Mol. Sci..

[38] Sachs J.R., Mayawala K., Gadamsetty S., Kang S.P., De Alwis D.P. (2016). Optimal dosing for targeted therapies in oncology: Drug development cases leading by example. Clin. Cancer Res..

[39] Thomford N.E., Senthebane D.A., Rowe A., Munro D., Seele P., Maroyi A., Dzobo K. (2018). Natural products for drug discovery in the 21st century: Innovations for novel drug discovery. Int. J. Mol. Sci..

[40] Schulze A., Harris A.L. (2012). How cancer metabolism is tuned for proliferation and vulnerable to disruption. Nature.

[41] Redza-Dutordoir M., Averill-Bates D.A. (2016). Activation of apoptosis signalling pathways by reactive oxygen species. Biochim. Biophys. Acta - Mol. Cell Res..

[42] Zorov D.B., Juhaszova M., Sollott S.J. (2014). Mitochondrial Reactive Oxygen Species (ROS) and ROS-Induced ROS Release. Physiol. Rev..

[43] Fulda S., Gorman A.M., Hori O., Samali A. (2010). Cellular stress responses: Cell survival and cell death. Int. J. Cell Biol..

[44] Lu L.Y., Ou N., Lu Q. (2013). Bin Antioxidant Induces DNA damage, cell death and mutagenicity in human lung and skin normal cells. Sci. Rep..

[45] Kang K.A., Piao M.J., Ryu Y.S., Hyun Y.J., Park J.E., Shilnikova K., Zhen A.X., Kang H.K., Koh Y.S., Jeong Y.J. (2017). Luteolin induces apoptotic cell death via antioxidant activity in human colon cancer cells. Int. J. Oncol..

[46] Kauffman M., Kauffman M., Traore K., Zhu H., Trush M., Jia Z., Li Y. (2016). MitoSOX-Based Flow Cytometry for Detecting Mitochondrial ROS. React. Oxyg. Species.

[47] Fukumura H., Sato M., Kezuka K., Sato I., Feng X., Okumura S., Fujita T., Yokoyama U., Eguchi H., Ishikawa Y. (2012). Effect of ascorbic acid on reactive oxygen species production in chemotherapy and hyperthermia in prostate cancer cells. J. Physiol. Sci..

[48] Ly J.D., Grubb D.R., Lawen A. (2003). The mitochondrial membrane potential (δψm) in apoptosis; an update. Apoptosis.

[49] Perelman A., Wachtel C., Cohen M., Haupt S., Shapiro H., Tzur A. (2012). JC-1: Alternative excitation wavelengths facilitate mitochondrial membrane potential cytometry. Cell Death Dis..

[50] Chen G., Yang Y., Xu C., Gao S. (2018). A flow cytometry-based assay for measuring mitochondrial membrane potential in cardiac myocytes after hypoxia/reoxygenation. J. Vis. Exp..

[51] Huang P., Feng L., Oldham E.A., Keating M.J., Plunkett W. (2000). Superoxide dismutase as a target for the selective killing of cancer cells. Nature.

[52] Surh Y.J. (2003). Cancer chemoprevention with dietary phytochemicals. Nat. Rev. Cancer.

[53] Martin-Cordero C., Jose Leon-Gonzalez A., Manuel Calderon-Montano J., Burgos-Moron E., Lopez-Lazaro M. (2012). Pro-Oxidant Natural Products as Anticancer Agents. Curr. Drug Targets.

[54] Fleury C., Mignotte B., Vayssière J.L. (2002). Mitochondrial reactive oxygen species in cell death signaling. Biochimie.

[55] Aggarwal V., Tuli H.S., Varol A., Thakral F., Yerer M.B., Sak K., Varol M., Jain A., Khan M.A., Sethi G. (2019). Role of reactive oxygen species in cancer progression: Molecular mechanisms and recent advancements. Biomolecules.

[56] Tang D., Kang R., Berghe T.V., Vandenabeele P., Kroemer G. (2019). The molecular machinery of regulated cell death. Cell Res..

[57] Singh R., Letai A., Sarosiek K. (2019). Regulation of apoptosis in health and disease: The balancing act of BCL-2 family proteins. Nat. Rev. Mol. Cell Biol..

[58] Hongmei Z. (2012). Extrinsic and Intrinsic Apoptosis Signal Pathway Review. Apoptosis Med..

[59] Kroemer G., Galluzzi L., Brenner C. (2007). Mitochondrial membrane permeabilization in cell death. Physiol. Rev..

[60] Green D.R., Kroemer G. (2004). The pathophysiology of mitochondrial cell death. Science (80-.).

[61] McArthur K., Whitehead L.W., Heddleston J.M., Li L., Padman B.S., Oorschot V., Geoghegan N.D., Chappaz S., Davidson S., Chin H.S. (2018). BAK/BAX macropores facilitate mitochondrial herniation and mtDNA efflux during apoptosis. Science (80-.).

[62] Vince J.E., De Nardo D., Gao W., Vince A.J., Hall C., McArthur K., Simpson D., Vijayaraj S., Lindqvist L.M., Bouillet P. (2018). The Mitochondrial Apoptotic Effectors BAX/BAK Activate Caspase-3 and -7 to Trigger NLRP3 Inflammasome and Caspase-8 Driven IL-1β Activation. Cell Rep..

[63] Luo J.L., Kamata H., Karin M. (2005). IKK/NF-κB signaling: Balancing life and death - A new approach to cancer therapy. J. Clin. Invest..

[64] Karin M., Cao Y., Greten F.R., Li Z.W. (2002). NF-κB in cancer: From innocent bystander to major culprit. Nat. Rev. Cancer.

[65] Kucharczak J., Simmons M.J., Fan Y., Gélinas C. (2003). To be, or not to be: NF-κB is the answer - Role of Rel/NF-κB in the regulation of apoptosis. Oncogene.

[66] Hayden M.S., Ghosh S. (2008). Shared Principles in NF-κB Signaling. Cell.

[67] Wu D.L., Liao Z.D., Chen F.F., Zhang W., Ren Y.S., Wang C.C., Chen X.X., Peng D.Y., Kong L.Y. (2019). Benzophenones from Anemarrhena asphodeloides Bge. Exhibit anticancer activity in HepG2 cells via the NF-κB signaling pathway. Molecules.

[68] Giridharan S., Srinivasan M. (2018). Mechanisms of NF-κB p65 and strategies for therapeutic manipulation. J. Inflamm. Res..

