# Supplementary material for: 2α-Hydroxyeudesma-4,11(13)-Dien-8β,12-Olide Isolated from Inula britannica Induces Apoptosis in Diffuse Large B-cell Lymphoma Cells
Source: Biomolecules. 2020 Feb 18;10(2):324. doi: 10.3390/biom10020324 (PMC7072307; doi:10.3390/biom10020324)
Supplement: Supplementary file 1 [file biomolecules-10-00324-s001.pdf]

biomolecules

MDPI

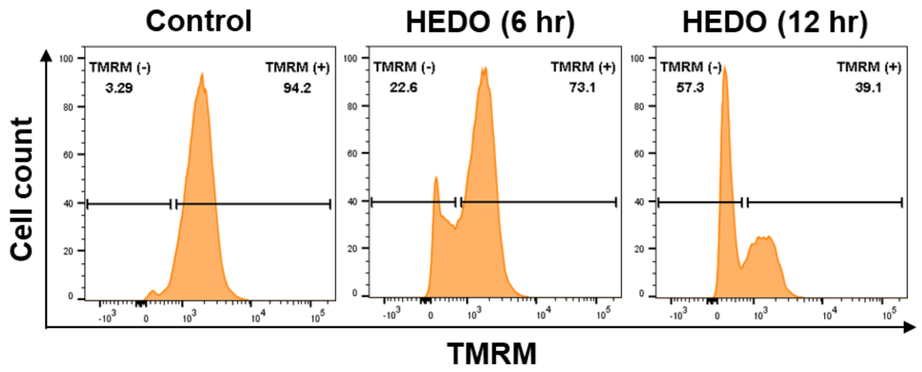

Figure S1. HEDO reduced the mitochondrial membrane potential within a short time in OCI-LY3 cells. Mitochondrial membrane potential of OCI-LY3 cells after 6 and 12 hours loaded with TMRM (100 nM), as detected by flow cytometry.

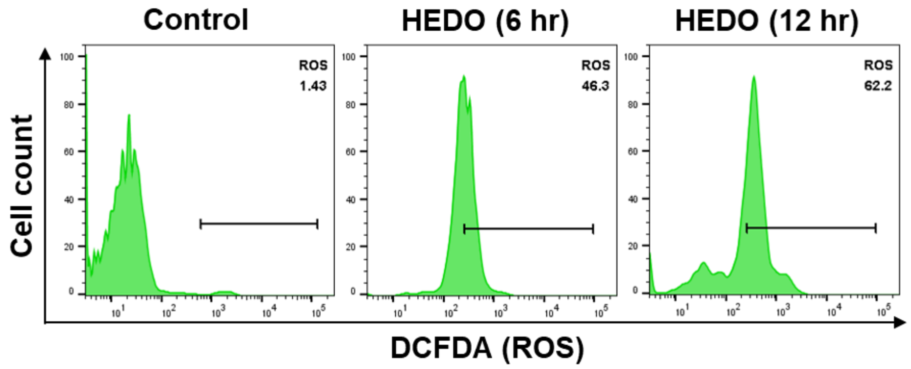

Figure S2. HEDO induced intracellular ROS levels within a short time in OCI-LY3 cells. Measurements of ROS levels in HEDO-treated OCI-LY3 cells after 6 and 12 hours, as detected by flow cytometry with DCFDA (1 μM).

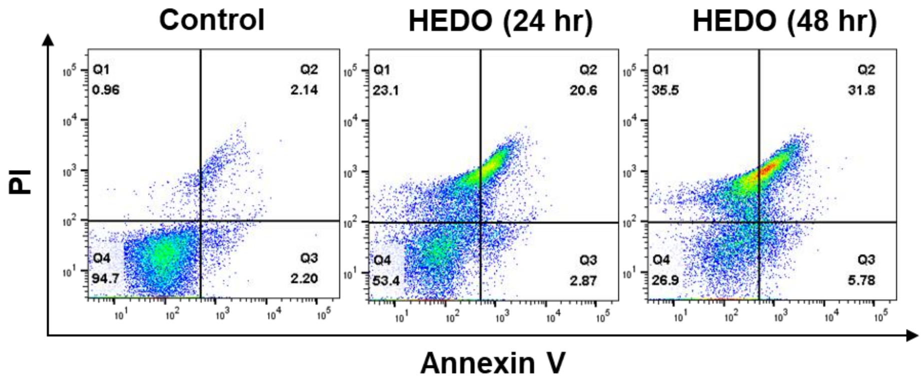

Figure S3. HEDO-induced apoptosis after 24 hr and 48 hr in OCI-LY3 cells. OCI-LY3 cells were untreated or treated with HEDO for 24 and 48 hours. Afterwards, apoptosis evaluation was performed via Annexin V-APC/PI double staining and flow cytometry.

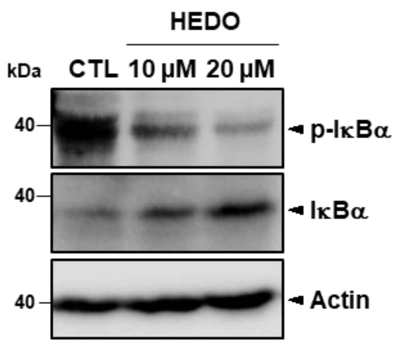

Figure S4. Effects of HEDO on NF-κB signaling in OCI-LY3 cells. Whole cell lysates were used to determine the expression levels of phosphorylated-I kappa B alpha (p-IκBα) and IκBα after treating the cells with HEDO.

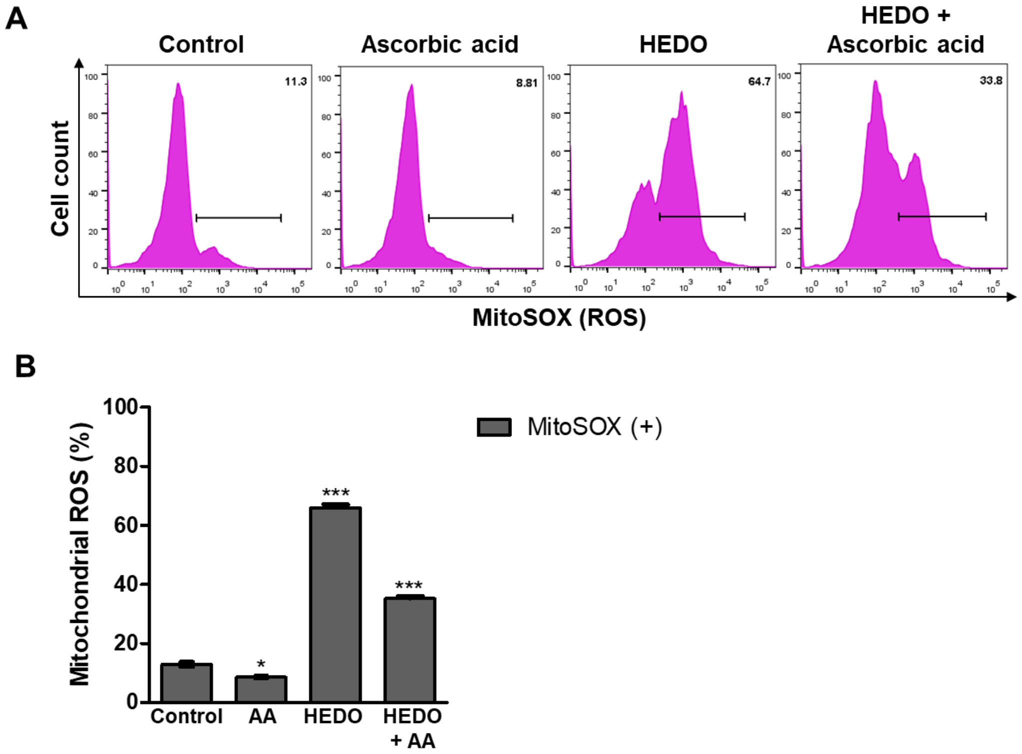

Figure S5. HEDO induced mitochondrial ROS levels in OCI-LY3 cells. (A) Measurements of mitochondrial ROS levels in HEDO-treated OCI-LY3 cells after 24 hours, as detected by flow cytometry with MitoSOX (5 μM). (B) Quantified mitochondrial ROS levels. Values indicate the means ± SEM. (n = 3, * $P < 0.05$, *** $p \leq 0.001$).

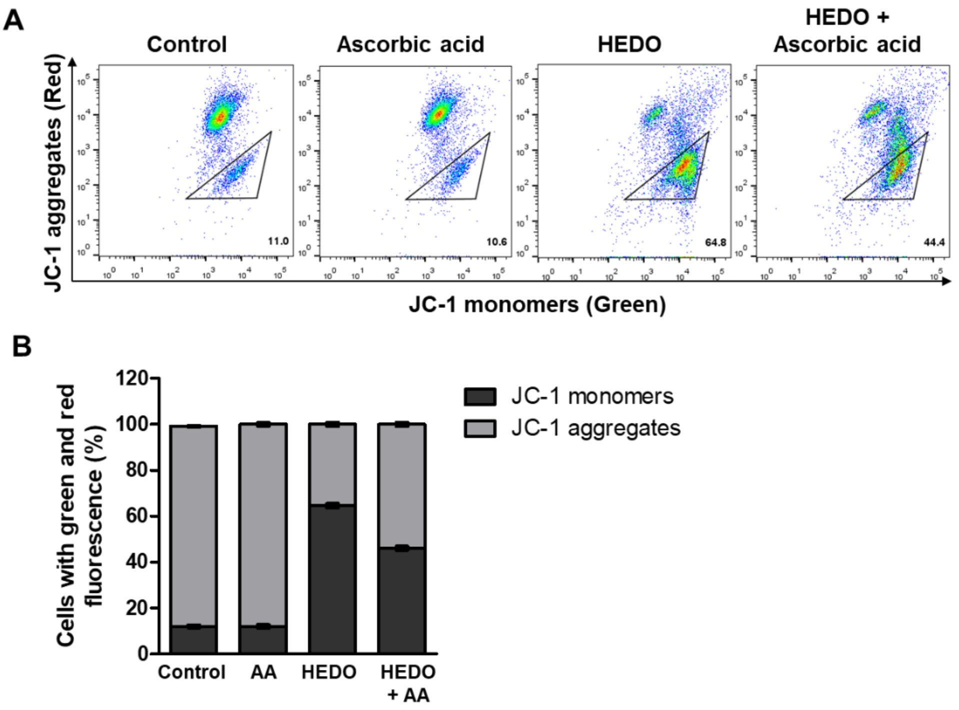

Figure S6. Mitochondrial membrane potential changes induced by HEDO in OCI-LY3 cells. (A) Measurements of mitochondrial membrane potential in HEDO-treated OCI-LY3 cells after 24 hours, as detected by flow cytometry with JC-1 (2 μM). (B) Quantified mitochondrial ROS levels.

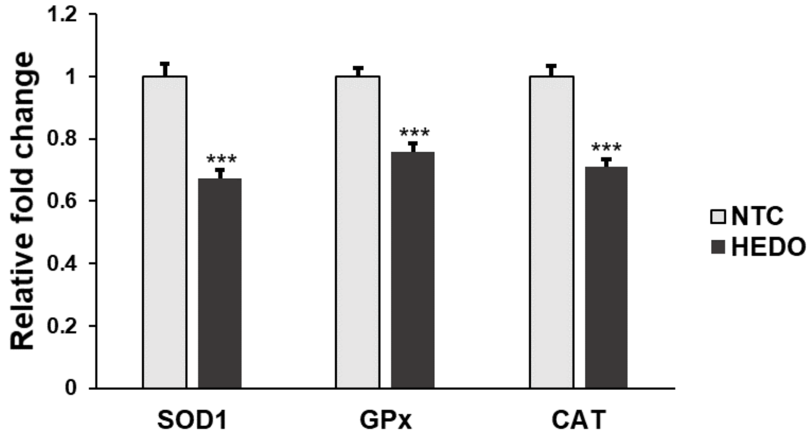

Figure S7. Effect of HEDO on the mRNA expression levels of involved antioxidants in OCI-LY3 cells. The qRT-PCR analysis results of the mRNA levels of SOD1, GPx, and CAT in the control and the HEDO (10 μM)-treated OCI-LY3 cells (n = 3, *** $p \leq 0.001$) were compared.
